# Assessment of factors influencing physicians’ intention to prescribe transfusion using the theory of planned behavior

**DOI:** 10.1186/s12913-023-09946-y

**Published:** 2023-09-08

**Authors:** Yu-Han Liao, Kung-Pei Tang, Chih-Yu Chou, Chien-Feng Kuo, Shin-Yi Tsai

**Affiliations:** 1 Department of Medicine, Mackay Medical College, New Taipei City, 252 Taiwan; 2 Department of Early Childhood & Family Education, National Taipei University of Education, Taipei City, Taiwan; 3 Department of Internal Medicine, Division of Infectious Diseases, Mackay Memorial Hospital, Taipei, Taiwan; 4 Department of Nursing, MacKay Junior College of Medicine, Nursing and Management, New Taipei City, 25245 Taiwan; 5 Department of Laboratory Medicine, Mackay Memorial Hospital, Taipei City 104, Taiwan; 6 Institute of Biomedical Sciences, Mackay Medical College, New Taipei City, Taiwan; 7 Institute of Long-Term Care, Mackay Medical College, New Taipei City, Taiwan; 8 Department of Health Policy and Management, Johns Hopkins Bloomberg School of Public Health, Johns Hopkins University, Baltimore, MD 21205 USA

**Keywords:** Transfusion, Physicians’ intention, Behavioral intention, Implementation intention

## Abstract

**Background:**

Blood shortage is a persistent problem affecting Taiwan’s health-care system. The theory of planned behavior (TPB) has been commonly used in studies of health advocacy. The purpose of this study was to develop a questionnaire measuring clinicians’ intention to prescribe transfusion based on the TPB.

**Method:**

A questionnaire comprising 15 items for assessing clinicians’ intention to prescribe blood transfusion was developed, and it collected demographic characteristics, tested patient blood management (PBM) and perceived knowledge of PBM. Furthermore, the questionnaire contained four subscales related to the TPB. A total of 129 clinicians participated in this pilot study between July and December2020. Item analysis and exploratory factor analysis were conducted to examine the validity and reliability of this measurement instrument.

**Results:**

The results indicated no statistically significant correlations between the demographic characteristics and PBM test scores. Regarding perceived knowledge, the results of a one-way analysis of variance revealed that the effect of age, hierarchy of doctors, and education level were significant. In terms of subjective norms, a significant effect on education level was noted [*t* (129) = 2.28, *p* < 0.05], with graduate school graduates receiving higher scores than college graduates. An analysis of variance demonstrated the effects of hierarchy, education level, and medical specialty on perceived behavioral control. The results of the regression analyses revealed that perceived knowledge (*β* = 0.32, *p* < 0.01) and subjective norms (*β* = 0.22, *p* < 0.05) were significantly related to clinicians’ behavioral intentions.

**Conclusions:**

This study revealed that factors affecting clinicians’ blood transfusion management can be explained using the TPB-based questionnaire. This study demonstrated that physicians’ perceptions of whether most people approve of PBM and their self-assessment of their PBM knowledge affect their intentions to proceed with PBM. According to this finding, a support system among physicians must be established and maintained to increase physicians’ confidence in promoting PBM.

**Supplementary Information:**

The online version contains supplementary material available at 10.1186/s12913-023-09946-y.

## Background

### The Demand of Patient Blood Management

Transfusion provides lifesaving therapeutic benefits for patients with anemia, thalassemia, sickle cell disease, and cancer. Anemia has become more important not only because of the increased prevalence (4.0% to 7.1% from 2003-2004 to 2011-2012 in United states) but also their association between chronic diseases such as heart failure, fibromyalgia, osteoporosis, chronic inflammation, Alzheimer’s disease, and so on [1–5]. Transfusion is a mainstay in the treatment of anemic patients. Blood transfusions are among the most common procedures performed in Taiwanese hospitals, with a total of 2,149,868 units of red blood cells (RBCs) used in 2015 [6]. According to American Red Cross, nearly 16 million blood components are transfused each year in the U.S.

Blood products are in high demand due to population aging and the increasing prevalence of cancers. The quantity of RBCs transfused has continued to rise over the last two decades, with most blood donors are aged between 30 and 50 years. Blood product availability continues to be a concern, especially during severe disasters or pandemics. The Taiwan Blood Services Foundation is raising awareness of the criticality of voluntary blood donation and reducing over transfusion and reviewing inappropriate transfusion practices are essential. Patient blood management (PBM) and its clinical application has gradually evolved and gained more attention over the last decades. Meeting patients’ needs individually through optimal evidence-based blood transfusion strategies is at the core of PBM and precision medicine.

Clinicians are accountable for ordering blood products and monitoring transfusion reactions. They have a key role in detecting the signs and symptoms of adverse reactions early and following standard transfusion practices to increase the quality of care. The decision to provide RBC transfusions should be based on clinical findings such as signs and symptoms, physical examinations, and laboratory data (e.g., hemoglobin values) [7].

Numerous guidelines have been developed to assist practitioners, such as “Clinical Practice Guidelines: Red Blood Cell Transfusion Thresholds and Storage from the American Association of Blood Banks” (AABB) and “the Maximum Surgical Blood Order Schedule.” The Taiwan Blood Services Foundation also provides data on optimal doses and indications of various blood components and the Taiwan Society of Blood Transfusion hosted a series of conferences in which they coordinated with different clinicians to share their experiences to advocate PBM during those years. Nevertheless, more than half of RBC transfusions may not have followed recommended practice indicating that the decision to provide transfusions is based on behavior and individual preferences than on scientific data, guidelines, or evidence-based risk versus benefit analysis [8, 9]. Potential barriers to applying clinical guidelines in physician practices include attitude, guideline unfamiliarity, lack of self-efficacy, outcome expectancy, inertia of previous practice and external barriers, such as financial resources, equipment, and time [10–12].

### Inappropriate transfusion

Although blood transfusion can be curative, certain risks and complications are associated with the treatment including bloodstream infections, transfusion-related acute lung injury, and coagulation abnormalities. Transfusion can also increase the length of hospital stay, infection rates, complications, and even mortality. Several studies have noted that transfusion does not improve the clinical outcomes of nonbleeding, critically ill patients with anemia. Inappropriate transfusion does not provide any benefits and may even lead to poor outcomes [13, 14].

Avoiding unnecessary blood transfusion not only reduces costs but also improves health-care outcomes [15]. For instance, patients with iron-deficiency anemia (IDA) who received iron supplementation generally had significantly lower hazard ratio for fibromyalgia than those who received blood transfusion alone [2]. The AABB provides education programs and publications for medical professionals at different levels worldwide, and most hospitals have their own employee training program. The effectiveness of interventions to reduce physicians’ levels of inappropriate transfusion was assessed in several systematic reviews [15–17]. Although the use of evidence- and theory-based approaches can help researchers understand the range of possible actions involved in intervention development and improve the effectiveness of interventions, thereby reducing costs and enhancing patients‘ prognosis, there still haven’t been relevant studies to compare the effectiveness and efficiency of existing interventions yet [18].

### Theory of planned behavior

Theory of planned behavior (TPB), a social cognitive theory linking beliefs to behavior [19], posits that individual behavior is driven by behavioral intention, a function of the following three components: attitude, subjective norms, and perceived behavioral control (PBC). Attitude toward behavior consists of outcome expectancy and behavioral belief, and subjective norms refer to the perceived social pressure from significant others. PBC relates to factors that can facilitate certain actions, including skills and abilities, time, financial resources, and the cooperation of others. PBC also refers to self-efficacy, a person’s belief in their capacity to successfully execute an action.

Various studies have used the TPB to analyze clinicians’ behavior and have examined the associations between perceived knowledge, tests of PBM background knowledge, and behavior [20–22].

Our study assessed the factors that affect clinicians’ behavior in relation to blood transfusion, with two hypotheses proposed. First, the TPB components and knowledge of blood transfusion are associated with clinicians’ behavior toward blood transfusion. Second, the demographic characteristics such as education level, hierarchy, and specialty also influence the clinicians’ behavior. The investigation of factors that affect PBM behavior would be valuable and informative for PBM policymakers.

## Method

Based on the TPB, we designed closed-ended questionnaires. The questionnaire design process followed a published reporting guideline (see Additional file 1) [23]. We involved nine steps in the development of this questionnaire: identifying the research purpose, defining the target respondents, selecting the questionnaire methods, designing the questionnaire outline, deciding on the question wording, sequencing the questions, subjecting the questionnaire to expert review, pretesting the questionnaire, and developing the final survey form [24].

This questionnaire collects the respondents’ background information and contains 64 items related to five theoretical domains: demographic characteristics, perceived knowledge and tests of blood transfusion knowledge, attitude toward deliberative transfusion prescription, subjective norms, and PBC [25]. Responses were scored on a 5-point Likert scale.

To ensure content validity, we invited 13 experts to form a panel and judge whether the questionnaire items adequately reflected the intended assessment and measured the items in the domain of interests. We provided three options (retain, revise, and remove) to the expert panel following their review of each initial item. A content validity index of greater than 0.8 ensured expert validity.

### Questionnaire pretest

We used the simple random sample method to obtain adequate samples in the population and collected data using online and paper questionnaires from July to December 2020. A total of 229 respondents participated in this survey. After removing respondents who were clerks, interns, nurse practitioners, and medical technologists and who therefore could not order blood transfusion for patients, we collected data from 129 respondents, from residents to attending physicians, in Taiwan.

We used item analysis and exploratory factor analysis (EFA) to explore the factor structure of the questionnaire. EFA is a dimension reduction method analyzing the relationship between the individual item variances and common variances shared between items. We applied Pearson correlation analysis to ensure that each item in the instrument was not highly correlated with each other. In addition, we verified the instrument’s reliability using Cronbach’s alpha. The indicators of item analysis included a critical ratio of greater than 0.3, an item to total correlation of greater than 0.4, a corrected item to total correlation of greater than 0.4, and the Cronbach’s alpha if an item was removed. We removed unsatisfactory items accordingly (questions 18, 27, and 28). The results are summarized in Table 1.

**Table 1:** Item analysis

Items	Differentiation	Congeniality			Action
	Critical ratio	Item-total correlation	Corrected item-total correlation	Cronbach’s α if item deleted	
14	4.78^**^	.359**	0.28	0.757	Reserved
15	3.94**	.408**	0.305	0.756	Reserved
16	3.8**	.401**	0.289	0.757	Reserved
17	6.27**	.515**	0.443	0.748	Reserved
18	2.4*	.317**	0.199	0.763	Deleted
19	5.9**	.533*	0.461	0.747	Reserved
20	5.83**	.497**	0.413	0.749	Reserved
21	5.48**	.53**	0.438	0.746	Reserved
22	4.37**	.412**	0.313	0.755	Reserved
23	6.92**	.523**	0.41	0.747	Reserved
24	3.91**	.393**	0.292	0.756	Reserved
25	7.98**	.657**	0.588	0.737	Reserved
26	4.06**	.395**	0.296	0.756	Reserved
27	1.69	.308**	0.16	0.77	Deleted
28	2.06*	.302**	0.14	0.775	Deleted
29	5.23**	.503**	0.419	0.748	Reserved
30	4.61**	.4**	0.316	0.755	Reserved
31	5.14**	.466**	0.396	0.751	Reserved
32	3.2**	.342**	0.237	0.76	Reserved
33	5.9**	.408**	0.349	0.755	Reserved
34	4.01**	.411**	0.302	0.756	Reserved

Cronbach’s α = 0.764, *p < .05. **p < .01

We conducted EFA to explore and verify the items in the questionnaire by identifying the common factors that explain the order and structure among measured variables. In this study, the results of the Kaiser–Meyer–Olkin (KMO) test and Bartlett’s test were significant for every dimension, indicating that all the variables were suitable for factor analysis. We performed EFA with orthogonal rotation on the questionnaire’s 21 items and then removed questions 26, 30, and 34 and performed factor analysis again; the results are presented as follows. After EFA, we extracted 15 items and grouped into four factors, which satisfied both the actual and desired purpose (Table 2).

**Table 2:** Factor loadings for the contributing items in the questionnaire

Item	Factor loading			
	Attitude	Subjective norm	Perceived behavioral control	Behavioral intention
14	0.674			
15	0.794			
16	0.545			
17	0.751			
19		0.88		
20		0.905		
21		0.823		
22			0.527	
23			0.834	
24			0.807	
25			0.727	
29				0.495
31				0.779
32				0.749
33				0.715

### Final survey development

The final questionnaire consisted of 28 items and was divided into three parts (see Additional file 2). The first part contained six items that collected the background information of the respondents such as age, gender, education level, and length of service. We used inferential statistics to describe the nature and distribution of the sample. The second part included three items that measured perceived knowledge and four items that measured PBM background knowledge. The third part consisted of TPB-based items, namely four items for attitudes toward prescribing deliberative transfusion, three items for subjective norms, four items for PBC, and four items for behavioral intention.

### Data collection and sample size

We collected data using online and paper questionnaires from July to December 2020 and used a convenience sample based on accessibility. We edited, coded, and tabulated the valid results from the questionnaire survey and excluded invalid questionnaires with incomplete data or the same answer to all questions.

Sample size calculations for the multiple regression analysis depended on the number of cases per predictor variable [26]. Five predictor variables were estimated, which required 146 respondents to test for multiple correlations and 116 to test individual predictor variables.

### Data analysis

We used SPSS software to compute descriptive statistics and performed an independent-samples *t* test to examine the differenced in education levels. We conducted a one-way analysis of variance (ANOVA) to compare the theoretical domains across age groups, hierarchies, and medical specialties and used linear regression analysis to explore the relationships between theoretical domains and behavioral intention to prescribe blood transfusions.

## Results

### Descriptive statistics

We collected 129 valid questionnaires before the cut-off date of December 3, 2021. Most respondents (Table 3) were men (72.1%, 93/129). The age groups (in years) were: 21–25 years = 26.4%, 26–30 years = 45.7%, 31–35 years = 10.1%, 36–40 years = 7.8%, and over 51 years = 7%. The specialties of the respondents were surgeons (34.1%), general practitioners (28.7%), and internal medicine physicians (24.8%).

**Table 3:** Demographic features of respondents in the study

Baseline characteristic	n	%
Gender		
Male	93	72.1
Female	36	27.9
Age		
21 ~ 25	34	26.4
26 ~ 30	59	45.7
31 ~ 35	13	10.1
36 ~ 40	10	7.8
41 ~ 45	4	3.1
46 ~ 50	0	0
≥ 51	9	7
Educational level		
College	66	51.2
Graduate school	63	48.8
Specialist		
General practitioner	37	28.7
Surgeon	44	34.1
Obstetrician	2	1.6
Pediatrician	4	3.1
Internal physician	32	24.8
Others	10	7.8
Doctor Hierarchy		
Resident	94	72.9
Chief resident	8	6.2
Junior attending physician	9	7
Senior attending physician	18	14

We used four questions tested the PBM background knowledge and three questions measured the perceived knowledge of blood transfusion practices. The participants scored an average of 2.78 out of 6 (standard deviation [SD] = 1.02) on the test. The mean scores for perceived knowledge, attitude, subjective norms, PBC, and behavioral intention were 2.51, 4.29, 4.20, 3.71, and 4.14, respectively (Table 4). The perceived knowledge was much lower than the other TPB components, indicating a low level of self-assessment of blood transfusion knowledge among physicians.

**Table 4:** Distribution of TPB variables on intentions to blood transfusion

Variable	Mean	SD
Test score of Background Knowledge about Blood Transfusion	2.78	1.02
Percieved Knowledge about Blood Transfusion	2.51	0.66
Attitude towards deliberative Transfusion Prescription	4.29	0.57
Subjective norms (SN)	4.2	0.72
Perceived behavioral control (PBC)	3.71	0.69
Behavior intention (BI)	4.14	0.52

The results of the multiple regression of clinicians’ behavioral intention. Perceived knowledge (*β* = 0.332, *p* < 0.01) and subjective norms (*β* = 0.169, *p* < 0.05) were significantly associated with clinicians’ behavioral intentions (Table 5).

**Table 5:** Regression coefficients of theoretical domain of TPB on clinicians’ behavior intention

Variables	*B*	*SE*	t	*p*	95% CI	
					LL	UL
Test score of Backgrond Knowledge about Blood Transfusion	0.067	0.041	1.641	0.103	-0.014	0.149
Perceived Knowledge about Blood Transfusion	0.258	0.07	3.678	.000***	0.119	0.397
Attitude towards deliberative Transfusion Prescription	0.054	0.077	0.704	0.483	-0.098	0.206
Subjective Norm	0.121	0.06	1.998	.048*	0.001	0.24
Percieved Behavour Control	0.047	0.07	0.673	0.502	-0.091	0.186
F	7.224***					
R	0.476					
*R* ^2^	0.227					
∆*R* ^2^	0.196					

*N* = 129. We examined the impact of theoretical domain of theory of planned behavior on clinicians’ behavior intention to prescribe transfusion. *p < .05. ***p < *.001*

The one-way ANOVA results showed significant differences in the perceived knowledge of blood transfusion by age group, hierarchy, and education level (Tables 6 and 7). Residents (mean [M] = 2.39, SD = 0.66) had significantly lower perceived knowledge than specialist physicians (M = 2.89, SD = 0.46, *p* = 0.006). Subjective norms did not correlate significantly with any demographic characteristics.

**Table 6:** Differences among theory of planned behavior constructs according to demographic characteristics

	Variable	Sum of Squares	df	Mean square	F	*p*
Age groups	tests	3.323	5	0.665	0.626	0.68
	KN	3.402	5	0.68	1.575	.038*
	AT	1.429	5	0.286	0.871	0.234
	SN	1.595	5	0.319	0.602	0.463
	PBC	1.627	5	0.325	0.668	0.648
Hierarchy	tests	3.517	3	1.172	1.124	0.342
	KN	5.289	3	1.763	4.3	.006**
	AT	0.123	3	0.041	0.123	0.946
	SN	0.354	3	0.118	0.222	0.881
	PBC	11.864	3	3.955	9.957	.000***
Specialty	tests	2.016	5	0.403	0.376	0.864
	KN	4.692	5	0.938	2.226	0.056
	AT	7.528	5	1.506	5.403	.000***
	SN	1.696	5	0.339	0.641	0.669
	PBC	12.629	5	2.526	6.355	.000***

*Test* Test score of Background Knowledge about Blood Transfusion, *KN* Percieved Knowledge about Blood Transfusion, *AT* Attitude towards deliberative Transfusion Prescription, *SN* subjective norms, *PBC* perceived behavioral control. *p< .05. **p< .01. ***p< .001

**Table 7:** Differences among theory of planned behavior constructs according to education level

Variable	College (*n* = 66)		Graduate school (*n* = 63)		df	t	*P*	95% CI	
	M	SD	M	SD				LL	UL
tests	2.83	1.02	2.73	1.04	127	-0.571	0.57	-0.46	0.25
KN	2.33	0.68	2.7	0.6	127	3.27	.001**	0.15	0.59
AT	4.27	0.59	4.31	0.55	127	0.48	0.63	-0.15	0.25
SN	4.12	0.77	4.28	0.66	127	1.25	0.21	-0.09	0.41
PBC	3.44	0.67	3.996	0.6	126.6	4.93	.000***	0.33	0.77

*Test* Test score of Background Knowledge about Blood Transfusion, *KN* Percieved Knowledge about Blood Transfusion, *AT* Attitude towards deliberative Transfusion Prescription, *SN* subjective norms, *PBC* perceived behavioral control. **p< .01. ***p< .001

The one-way ANOVA results also revealed significant differences in participants’ PBC across hierarchy, education level, and medical specialty. The residents’ PBC score (M = 3.53, SD = 0.68) was significantly lower than that of senior attending physicians (M = 4.15, SD = 0.41), junior attending physicians (M = 4.22, SD = 0.42), and chief residents (M = 4.31, SD = 0.51, *F* = 9.96, *p* =  < 0.001). Additionally, the mean PBC score of general practitioners (M = 3.35, SD = 0.65) was significantly lower than that of surgeons (M = 3.94, SD = 0.57) and interns (M = 4.02, SD = 0.65, *p* =  < 0.001).

## Discussion

Over the last two decades, the number of publications on factors affecting clinicians’ behavior toward blood transfusion has grown. Our TPB-based questionnaire was developed and verified to be reliable and valid through EFA and item analysis. The results revealed that clinicians’ behavior toward blood transfusion management can be explained using the TPB, with significant correlation among perceived knowledge, subjective norms, and behavioral intention observed. However, the score for PBM behavioral intention exhibited low variation (M = 4.14, SD = 0.52).

Regarding age group, hierarchy, and education level, clinicians’ evaluation of their PBM knowledge significantly differed. The higher the level of the respondents’ hierarchy was, the higher their score for perceived knowledge was. Additionally, regarding education level, the scores in the graduate school group (M = 2.7, SD = 0.60) were higher than those in the college group (M = 2.33, SD = 0.68). However, the correct answer rate for the test scores for PBM background knowledge was low in this questionnaire, with no significant correlation among demographic characteristics. One explanation is that the questions may have been too difficult; nevertheless, clinicians are relatively lacking in transfusion-related knowledge according to their identities. This result is consistent with the recent comment on the major obstacle to making transfusion practices more consistent and in line with published guidelines [27]. Furthermore, the Pearson correlation analysis indicated a weak linear relationship between tests of PBM background knowledge and perceived knowledge (correlation value = 0.02, *p* = 0.86). One explanation for this is that despite having similar test scores, attending physicians reported higher confidence in their knowledge levels compared with residents.

Subjective norms, such as those of medical laboratory scientists, physicians who specialize in transfusion medicine and transfusion committees, play key roles in the behavioral intention of clinicians. However, the results revealed no significant correlation between demographic characteristics and subjective norms.

The mean score for PBC was lower than that for attitude and subjective norms (M = 3.71, SD = 0.69). The results also showed significant correlations between PBC and education level, hierarchy, and medical specialty. This finding is expected when less experienced doctors, mainly general practitioners with bachelor's degree, have low levels of self-efficacy and external resources. Moreover, a lack of self-efficacy and familiarity may influence adherence to guideline recommendations in clinical situations [14]. Although demographic characteristics and PBC were significantly correlated, the effect of PBC on behavioral intention was low. They could be attributed to the strong influence of attending physicians’ advice on their residents’ decisions. Physicians are in a profession that traditionally functions in an extremely hierarchical fashion. Research on various medical professions has reported the negative impact of hierarchy, such as increased risk to patients, moral distress, and ethical dilemmas for residents as well as team functioning [28, 29]. The creation of learning environments that encourage residents to voice their opinions is crucial in medical education.

For future behavioral interventions, we recommend focusing on the influence of subjective norms and self-efficacy. Through the organization of multidimensional transfusion education based on real situations, knowledge, PBC, and subjective norms can be improved simultaneously. For example, combined conferences or simulation-based clinical rehearsals can increase clinicians’ familiarity with guidelines and transfusion details, which in turn increases their self-efficacy.

National policy should emphasize the importance of the medical education to raise awareness of blood transfusion. Further research on the effectiveness of continuing education on transfusion and behavioral intervention using the variable of learning hours instead of demographic characteristics is another method for evaluating clinicians’ behaviors.

Several studies have examined the effectiveness of behavioral intervention in modifying health-care practices [15]. Even though all interventions demonstrated varying degrees of effectiveness and the number of interventions implemented within a given study was not found to be associated with their effectiveness [30], the evidence level was low and the factors that affected behaviors changing still haven’t been investigated should be essential to propose promising interventions.

A decision support system may improve patient outcomes and safety by reducing inappropriate transfusion; for example, an electronic checklist could be used to assess the need for blood transfusion. The effectiveness of an electronic decision support blood order system in reducing inappropriate transfusion have been investigated and were included in NICE guideline, but the quality of evidence for all outcomes was low [31]. Along with the spectacular growth of artificial intelligence, the aspect of electronic decision support systems is worth exploring. 

## Conclusions

This study used a TPB-questionnaire to identify the factors affecting clinicians’ behavior in terms of blood transfusion. The main factors that influenced clinicians’ intention to prescribe blood transfusion in Taiwan were perceived knowledge and subjective norms.

Based on these results, we can determine which strategies to implement to increase awareness of the role and implications of PBM among clinicians. Moreover, we can propose promising strategies to persuade collaborators and trainees to apply blood transfusions appropriately.

Because of the wide coverage of national health insurance in Taiwan, the cost of blood products is not a major factor affecting clinicians’ behavior. Thus, we removed items related to this when conducting item analysis. Another factor that must be considered is that physicians work in a highly hierarchical fashion in Taiwan. Therefore, to further develop questionnaire items through transnational cooperation to compare the results from Taiwan with those from a different country would be valuable.

## Limitations

Our study referred to similar studies that used theoretical domain frameworks based on the TPB, but the questionnaire respondents did not represent all medical specialties, and the number of responses varied among each specialty. Accordingly, the results might not be fully generalized. Thus, collect comprehensive data from different specialties, staff, and geographical areas in Taiwan will be expected.

The sample size in this study was not large enough to perform structural equation modeling or confirmatory factor analysis. Consequently, further research analyzing more data is needed and collaborating with international experts would also allow the comparisons of the differences in behaviors affecting blood transfusion practices in a variety of clinical scenarios worldwide.

### Supplementary Information

**Additional file 1.**

**Additional file 2.**

## Data Availability

The data underlying the current study are available from the corresponding author upon reasonable request.
