# Supplementary material for: Assessment of factors influencing physicians’ intention to prescribe transfusion using the theory of planned behavior
Source: BMC Health Serv Res. 2023 Sep 8;23:973. doi: 10.1186/s12913-023-09946-y (PMC10492397; doi:10.1186/s12913-023-09946-y)
Supplement: Supplementary file 1 — Additional file 1. [file 12913_2023_9946_MOESM1_ESM.pdf]

Appendix 6: Considerations for the development and testing of a questionnaire

Item generation

- ☑ Conduct a literature review, use in-depth interviews or focus groups to generate items
- ❑ Sample for new questions until no new items are generated (redundancy)
- ☑ Group items with similar themes into domains or categories
- ☑ Generate a table of specifications

Item reduction

- ❑ Target ≤ 25 total items to answer your research question
- ☑ Use focus groups (with content experts) or external experts
- ☑ Consider statistical analyses (factor analysis) for longer and multidimensional surveys following pilot testing

Questionnaire formatting

Stem format

- ☑ Target total of ≤ 20 words for each question stem
- ☑ Use clear, succinct, unbiased, appropriate and nonjudgmental language suitable for the education level of respondents

Response formats

- ☑ Choose response format (binary, ordinal, nominal, interval, ratio) based on your stem format; this will determine the type of analysis
- ☑ Consider indeterminate response options ("uncertain," "don't know," "no opinion") to acknowledge uncertainty or indecisiveness of respondent
- ❑ Consider "other" response options for respondents to allow for unanticipated responses, identify new issues or elaborate upon responses to closed questions

Questionnaire composition

- ☑ Include a cover letter
- ☑ Highlight the rationale for the study and how respondents were chosen
- ☑ Consider placing the demographic questions at the end of the questionnaire if the questions are of a sensitive nature

Pre-testing

- ☑ Pre-test the full draft questionnaire and the cover letter
- ☑ Use personal interviews or focus groups (include experts/nonexperts) to pre-test
- ☑ Consider potential users of the information generated from the results of your survey, research colleagues or a cross-section of potential respondents to pre-test
- ☑ Evaluate each question and determine a course of action (i.e., accept original question, accept question with a change in its meaning, change question but retain meaning, eliminate question, develop new question)

Pilot testing

- ☑ Pilot test the penultimate version of the questionnaire to determine whether you have optimized the question order, relevance of the included questions and general flow of the questionnaire
- ☑ Ask this group of respondents to asses the length of questionnaire and ease with which they completed the questionnaire
- ☑ Consider using factor analysis to further reduce items following pilot testing (at least 5 respondents per candidate item required)

Clinical sensibility testing

- ❑ Conduct clinical sensibility testing to assess the comprehensiveness, clarity and face validity of the questionnaire
- ☑ Administer a 1-page assessment sheet to respondents with items posed as questions with either Likert scale or nominal response formats

Reliability

- ☑ Choose the specific reliability assessment (test–retest, interrater, internal consistency) based on the survey's objective and the format of the responses
- ❑ To assess test–retest reliability, administer the final questionnaire to selected respondents representing the sampling frame on 2 occasions at least 2–6 weeks apart

Validity

- ☑ Assess content validity by asking a content expert
- ❑ If you have created a *table of specifications*, ask a content expert to review your table
- ☑ Ask a content expert to assess construct validity to determine whether key constructs underlying your study question have been addressed by the questions

Appendix to Burns KEA, Duffett M, Kho M, et al.; ACCADEMY Group. A guide for the design and conduct of self-administered surveys of clinicians. *CMAJ* 2008;179(3):245-52.
