# Supplementary material for: Assessment of factors influencing physicians’ intention to prescribe transfusion using the theory of planned behavior
Source: BMC Health Serv Res. 2023 Sep 8;23:973. doi: 10.1186/s12913-023-09946-y (PMC10492397; doi:10.1186/s12913-023-09946-y)
Supplement: Supplementary file 2 — Additional file 2. [file 12913_2023_9946_MOESM2_ESM.pdf]

1. Gender: ☐ Male ☐ Female
2. Age: ☐20~25 ☐26~30 ☐31~35 ☐36~40 ☐41~50 ☐51~60 ☐>61
3. Education level: ☐ Diploma ☐ Bachelor’s degree ☐ Master’s ☐ Ph.D
4. Hospital position and years experience (please specify):<br>☐Specialist physician (>5 years)<br>☐Junior specialist physician (<5 years)<br>☐Chief Resident doctor<br>☐Resident doctor<br>☐Intern doctor ☐Clerk<br>☐Nurse practitioner<br>☐Lab technician<br>The number of years experience after you got license: ____
5. Which specialty are you currently working in (please specify):<br>☐Internal medicine：______________ ☐Surgery:________________<br>☐Pediatrics: _________ ☐Obstetrics-gynecology: _____________<br>☐Other clinical specialty: _____________ ☐Post-graduate year (PGY)<br>☐Internship ☐Clerkship
6. Work location (please check all that applies):<br>☐Ward □Intensive Care Unit ☐Emergency department ☐Outpatient clinic ☐Operating theater<br>☐Recovery room ☐ Haemodialysis Unit ☐Chemotherapy unit ☐Laboratory<br>☐Other: ___________
The following questions are based on your understanding of blood product transfusion policy and processes, please choose the most appropriate response.
**7. I understand the whole process of product transfusion, from the physician’s order to patient transfusion.**<br>□ Strongly agree<br>□ Agree<br>□ Neither agree nor disagree<br>□ Disagree<br>□ Strongly disagree
**8. I understand that under some circumstances, patients’ blood products may not need to undergo crossmatching test.**<br>□ Strongly agree<br>□ Agree<br>□ Neither agree nor disagree<br>□ Disagree<br>□ Strongly disagree
**9. I understand the Patient Blood Management Checklist.**<br>□ Strongly agree<br>□ Agree<br>□ Neither agree nor disagree<br>□ Disagree<br>□ Strongly disagree
**10. At what platelet count would you provide a prophylactic platelet transfusion for patients with hematologic disorder (e.g., AML)**<br>□ 5,000/ul<br>□ 10,000/ul<br>□ 20,000/ul<br>□ 50,000/ul<br>□ 100,000/ul

11. What is the acceptable range for Blood to Plasma ratio?
□Less than 1 □ Maintained at 1~2 □Greater than 2 □Unaware

12. The cost of transfusion a unit of packed RBC.
□ NTD 45
□ NTD 75
□ NTD 450
□ NTD 750

13. The cost of transfusion a unit of leukocyte poor plateletpheresis.
□ NTD 450
□ NTD 750
□ NTD 4500
□ NTD 7500

The following questions are based on your understanding of blood product transfusion policy and processes, please choose the most appropriate response.

14. It is important for doctors to regulate the amount of blood product transfusion orders.
□ Strongly agree
□ Agree
□ Neither agree nor disagree
□ Disagree
□ Strongly disagree

15. To prevent a shortage of blood products, I think it is necessary to control the number of blood product requests.
□ Strongly agree
□ Agree
□ Neither agree nor disagree
□ Disagree
□ Strongly disagree

16. In general, when managing patients with slight lower hemoglobin (Hb) levels compared with the trigger for required transfusion, I would observe symptoms/signs and keep monitoring the Hb prior to prescribing the blood transfusion directly.
□ Strongly agree
□ Agree
□ Neither agree nor disagree
□ Disagree
□ Strongly disagree

17. Most literature advocates for stringent regulations regarding requesting blood components.
□ Strongly agree
□ Agree
□ Neither agree nor disagree
□ Disagree
□ Strongly disagree

18. When ordering blood products, I may be influenced by advice from physicians who specialize in blood banking/transfusion medicine.
□ Strongly agree
□ Agree
□ Neither agree nor disagree
□ Disagree
□ Strongly disagree

19. When ordering blood products, I may be influenced by advice from medical technicians.
□ Strongly agree
□ Agree
□ Neither agree nor disagree
□ Disagree
□ Strongly disagree

**20. When ordering blood products, I may be influenced by advice from the transfusion committee.**<br>□ Strongly agree<br>□ Agree<br>□ Neither agree nor disagree<br>□ Disagree<br>□ Strongly disagree
**21.When ordering blood products, I may be influenced by advice from clinical guideline and important publications.**<br>□ Strongly agree<br>□ Agree<br>□ Neither agree nor disagree<br>□ Disagree<br>□ Strongly disagree
**22. To manage patients with borderline hemoglobin level, I am confident in myself to watch his/her condition to assess if there's an unmet need for transfusion.**<br>□ Strongly agree<br>□ Agree<br>□ Neither agree nor disagree<br>□ Disagree<br>□ Strongly disagree
**23. To manage patients with borderline hemoglobin level, I am confident in myself to assess the type and the number of units of blood products required.**<br>□ Strongly agree<br>□ Agree<br>□ Neither agree nor disagree<br>□ Disagree<br>□ Strongly disagree
**24. To manage patients with borderline hemoglobin level, I would consider alternatives rather than requesting for blood transfusion.**<br>□ Strongly agree<br>□ Agree<br>□ Neither agree nor disagree<br>□ Disagree<br>□ Strongly disagree
**25. It is possible for me to control the number of blood product requests I make.**<br>□ Strongly agree<br>□ Agree<br>□ Neither agree nor disagree<br>□ Disagree<br>□ Strongly disagree
**26. I assess the patients' clinical status prior to ordering blood products.**<br>□ Strongly agree<br>□ Agree<br>□ Neither agree nor disagree<br>□ Disagree<br>□ Strongly disagree
**27. To determine estimated blood loss, I consider the patient's clinical symptoms, laboratory data or images.**<br>□ Strongly agree<br>□ Agree<br>□ Neither agree nor disagree<br>□ Disagree<br>□ Strongly disagree

28. I order blood components based on patients' clinical indications and circumstance.

□ Strongly agree
□ Agree
□ Neither agree nor disagree
□ Disagree
□ Strongly disagree
